# A New Model of Spontaneous Colitis in Mice Induced by Deletion of an RNA m^6^A Methyltransferase Component METTL14 in T Cells

**DOI:** 10.1016/j.jcmgh.2020.07.001

**Published:** 2020-07-04

**Authors:** Thomas X. Lu, Zhong Zheng, Linda Zhang, Hui-Lung Sun, Marc Bissonnette, Haochu Huang, Chuan He

**Affiliations:** 1 Section of Gastroenterology, Hepatology, and Nutrition, University of Chicago Medicine, Chicago, Illinois; 2 Department of Chemistry, University of Chicago, Chicago, Illinois; 3 Department of Biochemistry and Molecular Biology, University of Chicago, Chicago, Illinois; 4 Genentech, South San Francisco, California; 5 Howard Hughes Medical Institute, Chicago, Illinois

**Keywords:** Inflammatory Bowel Disease, RNA m^6^A Methylation, Mouse Colitis Model, Regulatory T Cells, FACS, fluorescence-activated cell sorter, IBD, inflammatory bowel disease, IFNγ, interferon gamma, IL, interleukin, iT_reg_, induced regulatory T, m^6^A, *N*^6^-methyladenosine, mRNA, messenger RNA, OTU, operational taxonomic unit, T_reg_, regulatory T cell, TNFα, tumor necrosis factor alpha

## Abstract

**Background and aims:**

Mouse models of colitis have been used to study the pathogenesis of inflammatory bowel disease (IBD) and for pre-clinical development of therapeutic agents. Various epigenetic pathways have been shown to play important regulatory roles in IBD. Reversible *N*^6^-methyladenosine (m^6^A) methylation represents a new layer of post-transcriptional gene regulation that affects a variety of biological processes. We aim to study how deletion of a critical component of m^6^A writer complex, METTL14, in T cells affects the development of colitis.

**Methods:**

Conditional *Mettl14* was lineage specifically deleted with CD4-regulated Cre in T cells. Colitis phenotype was determined by H&E staining, colon weight-to-length ratio and cytokine expression. We additionally utilized T cell transfer model of colitis and adoptive transfer of regulatory T cells. Mice were treated with antibiotics to determine if the colitis could be attenuated.

**Results:**

METTL14 deficiency in T cells induced spontaneous colitis in mice. This was characterized by increased inflammatory cell infiltration, increased colonic weight-to-length ratio and increased Th1 and Th17 cytokines. The colitis development was due to dysfunctional regulatory T (T_reg_) cells, as adoptive transfer of WT T_reg_ cells attenuated the colitis phenotype. The METTL14-deficient T_reg_ cells have decreased RORγt expression compared with WT controls. METTL14 deficiency caused impaired induction of naïve T cells into induced T_reg_ cells. Antibiotic treatment notably attenuated the colitis development.

**Conclusion:**

Here we report a new mouse model of spontaneous colitis based on perturbation of RNA methylation in T cells. The colitis is T cell-mediated and dependent on the microbiome. This model represents a new tool for elucidating pathogenic pathways, studying the contribution of intestinal microbiome and preclinical testing of therapeutic agents for inflammatory bowel disease.

**Synopsis**

We have found that deletion of RNA methylation writer METTL14 in T cells induced spontaneous colitis in mice. This is characterized by a Th1/Th17 phenotype. The colitis development was due to dysfunctional regulatory T cells and is dependent on the microbiome.

The incidence and prevalence of inflammatory bowel disease (IBD) are increasing worldwide in association with increasing urbanization.^1^ Mouse models of colitis have been used to study the pathogenesis of IBD and for preclinical development of new therapeutic modalities. Multiple experimental models of IBD have been developed. While no single model recapitulates the full spectrum of human IBD, the spectrum of models provides insights into various aspects of the disease.^2^

Reversible messenger RNA (mRNA) *N*^6^-methyladenosine (m^6^A) methylation serves as an additional layer of post-transcriptional regulation.^3^^,^^4^ This abundant mRNA modification is installed by a methyltransferase complex with the catalytic core containing a heterodimer of METTL3 (methyltransferase like 3) and METTL14 (methyltransferase like 14), which also binds an accessory protein WTAP.^5^, ^6^, ^7^ Global deletion of *Mettl14* leads to embryonic lethality early in gestation.^8^ Deletion of *Mettl3* or *Mettl14* have been shown to impact a range of developmental processes.^4^^,^^9^, ^10^, ^11^, ^12^, ^13^, ^14^, ^15^

T cells have been shown to be integral in the pathogenesis of IBD. Several currently approved biologic therapies, including anti-integrin therapy and anti-IL12/23 therapy, act by preventing recruitment or activation of T cells.^16^ It has been reported that a deletion of RNA methylation writer enzyme *Mettl3* in T cells leads to dysfunction in both naïve T cells and T_reg_ cells, with naïve T cells losing their ability to induce inflammation and T_reg_ cells losing their immune suppressive capacity.^14^^,^^17^ Using a conditional genetic deletion approach, we selectively deleted *Mettl14*, another component of the methyltransferase complex key to the methylation, in T cells. This leads to a global loss of mRNA m^6^A in T cells. Here, we show that deletion of *Mettl14* in T cells induces development of spontaneous colitis in mice.

## Results

### *CD4-Cre*^+/Tg^*Mettl14*^FL/FL^ Conditional Knockout Mice Develop Spontaneous Colitis

**Figure 1:**
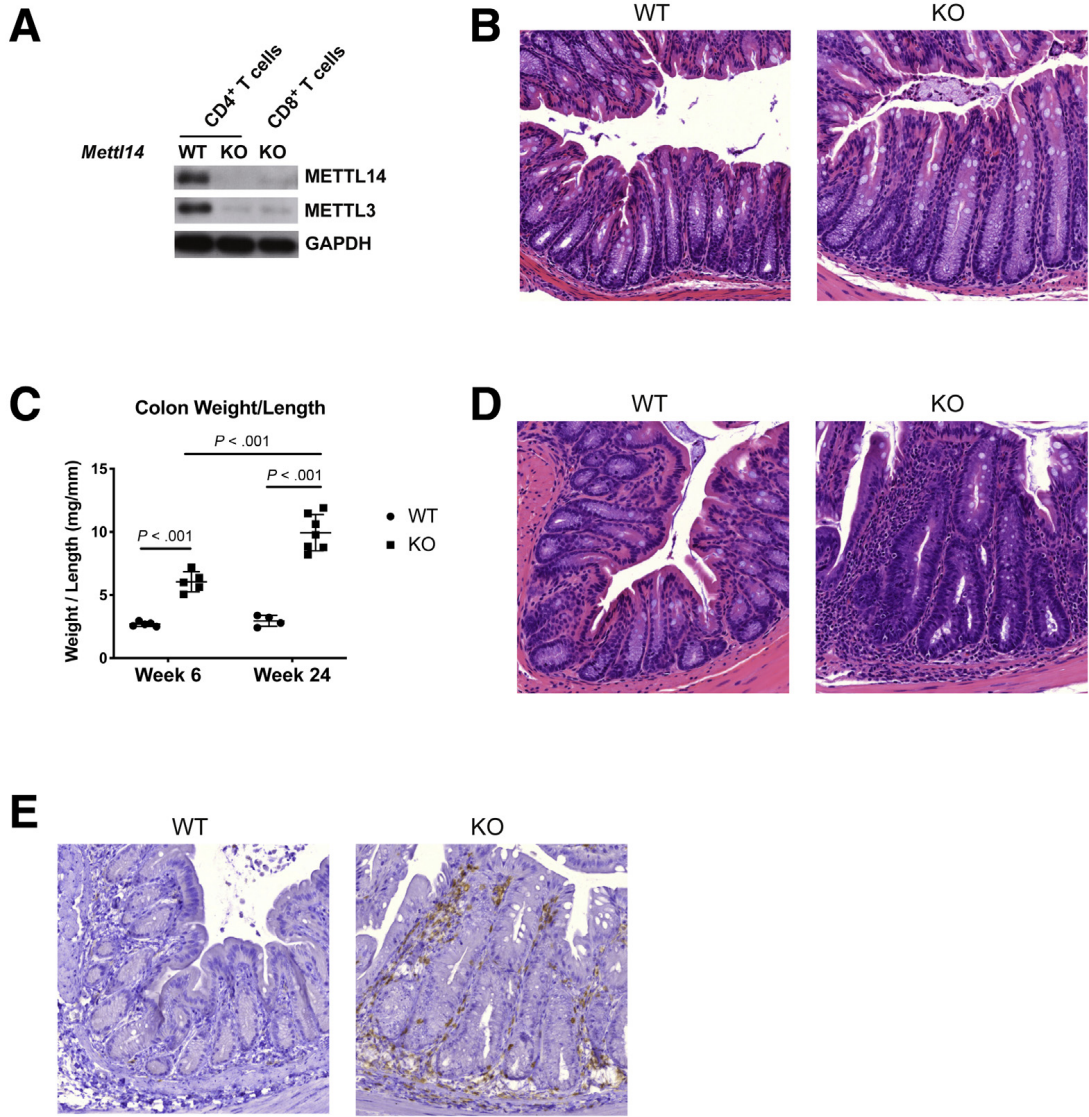
**Colonic weight-to-length ratios and histology of *CD4-Cre*^+/Tg^*Mettl14*^FL/FL^ conditional knockout mice.** (*A*) CD4^+^ and CD8^+^ T cells were isolated by FACS sorting and analyzed by Western blot for METTL14 and associated METTL3 expression. GAPDH is used as an internal control. (*B*) Hematoxylin and eosin stain of colon at 4 weeks of age. (*C*) Colonic weight-to-length ratio of mice at 6 and 24 weeks of age. Data are represented as mean ± SD. (*D*) Hematoxylin and eosin stain of colon at 24 weeks of age. (*E*) Anti-CD4 immunohistochemistry staining of colon cross-section at 24 weeks of age. n = 4–8 mice per group. Data representative of 2 independent experiments with similar results. WT indicates wild-type littermate control. KO indicates *CD4-Cre*^+/Tg^*Mettl14*^FL/FL^ conditional knockout mouse.

We generated the *CD4-Cre*^+/Tg^ *Mettl14*^FL/FL^ conditional knockout mice by crossing the *CD4-Cre*^+/Tg^ mice with the *Mettl14*^FL/FL^ mice. In these mice, the *Mettl14* allele is selectively deleted in T cells. Analysis of the T cells by Western blot showed absence of METTL14 protein and significantly decreased expression of the associated METTL3 protein (Figure 1*A*). The mice null for T cell METTL14 are normal at 4 weeks of age with normal colonic histology (Figure 1*B*). By 6 weeks of age, the *CD4-Cre*^+/Tg^ *Mettl14*^FL/FL^ mice developed colitis characterized by increased colonic weight-to-length ratio (Figure 1*C*). The colitis became progressively more severe with a much higher colonic weight-to-length ratio by 24 weeks compared with 6 weeks (Figure 1*C*). Histology showed significant mucosal damage and inflammation characterized by disruption of crypt architecture, crypt branching, crypt abscess formation, marked increase in mucosal thickness, and lymphocyte infiltration in the epithelial and submucosal layers in the *CD4-Cre*^+/Tg^ *Mettl14*^FL/FL^ mice (Figure 1*D*). Most of the lymphocytes were CD4^+^ as seen on immunochemistry staining (Figure 1*E*).

### Colitis in *CD4-Cre*^+/Tg^*Mettl14*^FL/FL^ Conditional Knockout Mice Showed a Th1/Th17 Predominant Phenotype

**Figure 2:**
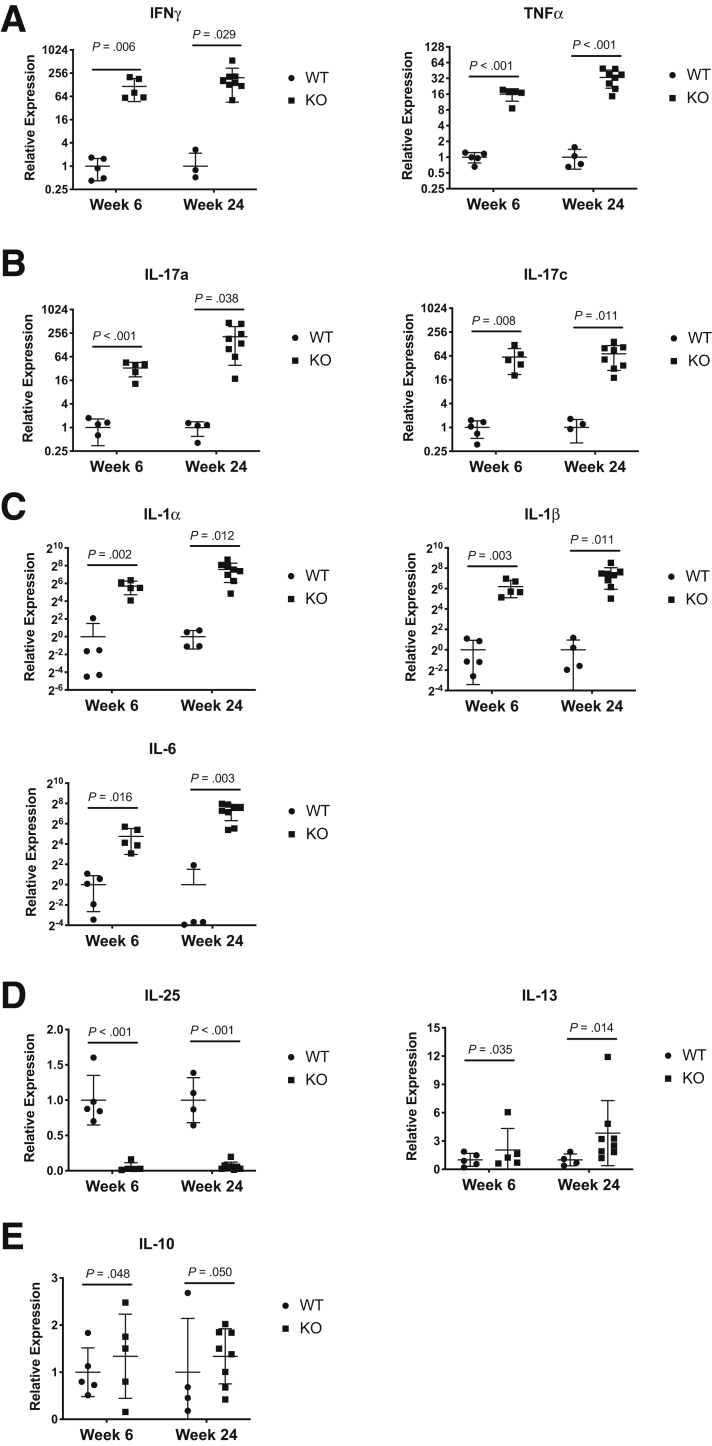
**Cytokine expression profile of *CD4-Cre*^+/Tg^*Mettl14*^FL/FL^ Conditional knockout mice.** Relative expression of (*A*) Th1 cytokines IFNγ and TNFα; (*B*) Th17 cytokines IL-17a and IL-17c; (*C*) cytokines associated with inflammation including IL-1α, IL-1β, and IL-6; (*D*) Th2-potentiating cytokine IL-25 and Th2 cytokine IL-13; and (*E*) inhibitory cytokine IL-10. WT indicates wild-type littermate control. KO indicates *CD4-Cre*^+/Tg^*Mettl14*^FL/FL^ conditional knockout. Log_2_ scale was used on the y-axis for panels *A*–*C*; linear scale was used on the y-axis for panels *D* and *E*. Data are represented as mean ± SD. n = 4–8 mice per group. Data representative of 2 independent experiments with similar results.

To determine whether the colitis was mediated by a Th1 or Th2 or Th17 response, we measured the cytokine profile from colonic epithelial scrapping of *CD4-Cre*^+/Tg^ *Mettl14*^FL/FL^ conditional knockout mice compared with littermate control mice. *CD4-Cre*^+/Tg^ *Mettl14*^FL/FL^ conditional knockout mice had a marked increase in Th1 cytokines characterized by increased interferon gamma (IFNγ) and tumor necrosis factor alpha (TNFα) (Figure 2*A*). The *CD4-Cre*^+/Tg^ *Mettl14*^FL/FL^ conditional knockout mice also exhibited a marked increase in Th17 cytokines characterized by increased interleukin (IL)-17a and IL-17c (Figure 2*B*). Other cytokines associated with inflammation were also increased including increased IL-1α, IL-1β, and IL-6 (Figure 2*C*). In contrast, the level of Th2-potentiating cytokine IL-25 was significantly reduced, and the level of Th2 cytokine IL-13 was similar between the *CD4-Cre*^+/Tg^ *Mettl14*^FL/FL^ conditional knockout mice and littermate control mice (Figure 2*D*). The level of inhibitory cytokine IL-10 was similar between the *CD4-Cre*^+/Tg^ *Mettl14*^FL/FL^ conditional knockout mice and littermate control mice (Figure 2*E*).

### *Mettl14*-Deficient T_reg_ Cells Are Unable to Suppress Naïve T Cell–Induced Colitis

**Figure 3:**
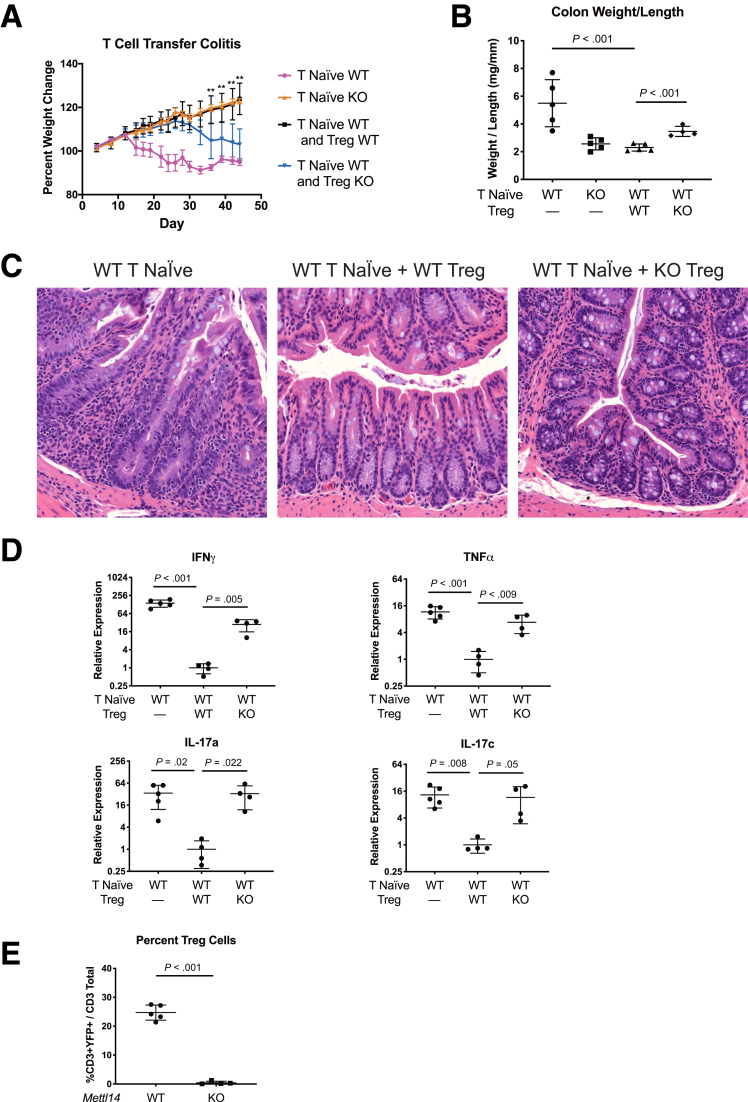
***Mettl14*-deficient T_reg_ cells are unable to suppress naïve T cell–induced colitis in *Rag1*^*–/–*^ adoptive transfer T cell model of colitis.** WT naïve T cells, *Mettl14*^–/–^ naïve T cells, WT naïve T cells + WT T_reg_ cells, or WT naïve T cells + *Mettl14*^–/–^ T_reg_ cells were transferred to *Rag1*^*–/–*^ mice. (*A*) Weight change. ∗∗*P <* .01 comparing mice that received WT naïve T cells + WT T_reg_ cells vs WT naïve T cells + *Mettl14*^–/–^ T_reg_ cells. (*B*) Colon weight-to-length ratio. (*C*) Hematoxylin and eosin stain of colon. (*D*) Relative expression of Th1 cytokine IFNγ/TNFα and Th17 cytokine IL-17a/IL-17c. Log_2_ scale was used on the y-axis. (*E*) Flow cytometry analysis of percent of T_reg_ cell in mesenteric lymph nodes. WT indicates wild-type littermate control. KO indicates *CD4-Cre*^+/Tg^*Mettl14*^FL/FL^ conditional knockout. Data are represented as mean ± SD; n = 4–5 mice per group. Data representative of 2 independent experiments with similar results.

The colitis seen in the *CD4-Cre*^+/Tg^ *Mettl14*^FL/FL^ conditional knockout mice could be due to either hyperactive proinflammatory T cells or defective T_reg_ cells that are unable to maintain homeostasis and prevent spontaneous inflammation. To test these hypotheses, we utilized a T cell transfer colitis model where naïve T cells were transferred either alone or together with T_reg_ cells into lymphopenic *Rag1*^–/–^ mice. It has been reported that when wild-type (WT) naïve T cells are transferred into the lymphopenic *Rag1*^–/–^ mice, the *Rag1*^–/–^ mice develop colitis after 4–8 weeks. Co-transferring T_reg_ cells has been shown to suppress the naïve T cell–induced colitis in this model.^18^ We found that transferring WT naïve T cells into *Rag1*^–/–^ mice induced colitis in the *Rag1*^–/–^ mice characterized by progressive weight loss over time (Figure 3*A*). However, transferring *Mettl14*^–/–^ naïve T cells was unable to induce weight loss in the recipient *Rag1*^–/–^ mice (Figure 3*A*). When WT naïve T cells were transferred with WT T_reg_ cells into *Rag1*^–/–^ mice, the WT T_reg_ cells were able to completely suppress the naïve T cell–induced colitis with no weight loss seen after 7 weeks (Figure 3*A*). When we transferred *Mettl14*-deficient T_reg_ cells with the WT naïve T cells into the *Rag1*^–/–^ mice, the *Mettl14*-deficient T_reg_ cells were unable to suppress naïve T cell–induced colonic inflammation. The mice developed progressive weight loss (Figure 3*A*), with increased colonic weight-to-length ratio compared with mice that received wildtype T_reg_ cells (Figure 3*B*). These data support that the colitis phenotype seen in the *CD4-Cre*^+/Tg^ *Mettl14*^FL/FL^ conditional knockout mice was predominantly due to dysfunctional T_reg_ cells.

We subsequently focused our analysis on mice that were co-transferred with T_reg_ cells. Histology showed that mice that were co-transferred with WT T_reg_ cells developed no inflammation while marked inflammation was seen in the mice that were co-transferred with *Mettl14*-deficient T_reg_ cells (Figure 3*C*). Cytokine analysis showed that while WT T_reg_ cells were able to suppress naïve T cell–induced Th1 cytokines IFNγ/TNFα and Th17 cytokines IL-17a/IL-17c, the *Mettl14*-deficient T_reg_ cells were unable to suppress naïve T cell–induced Th1 and Th17 cytokine production (Figure 3*D*). Flow cytometry analysis showed that mice received *Mettl14*-deficient T_reg_ cells have a marked decrease of T_reg_ cells in the mesenteric lymph nodes compared with mice that received WT T_reg_ cells (Figure 3*E*).

### Adoptive Transfer of T_reg_ Cells Attenuates the Colitis in *CD4-Cre*^+/Tg^*Mettl14*^FL/FL^ Conditional Knockout Mice

**Figure 4:**
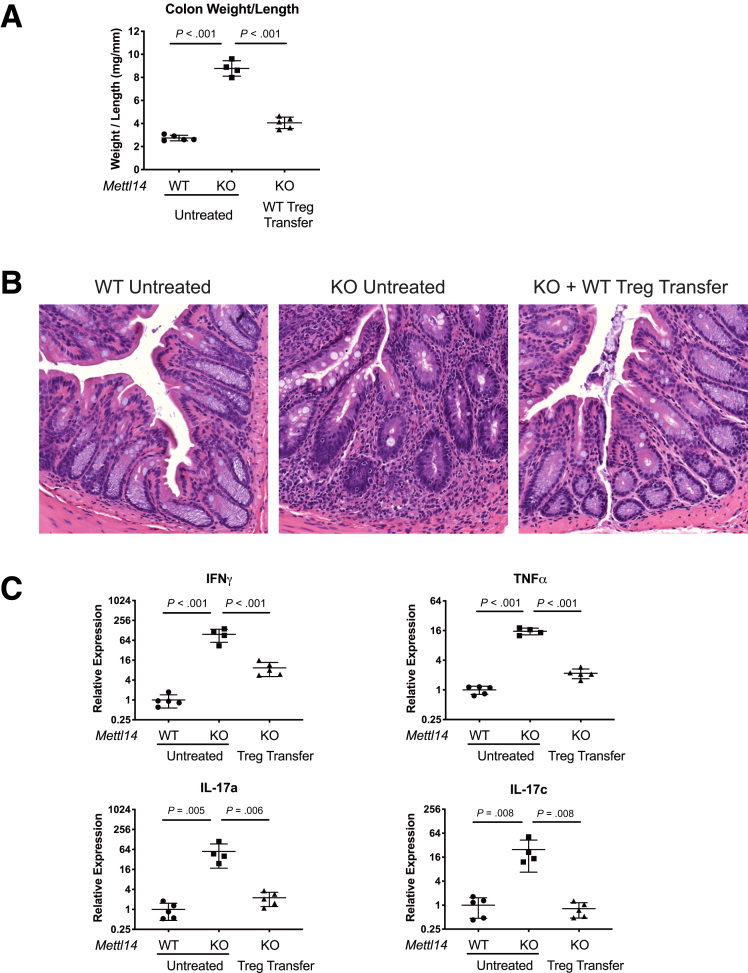
**Adoptive transfer of WT T_reg_ cells attenuates the colitis in *CD4-Cre*^+/Tg^*Mettl14*^FL/FL^ conditional knockout mice.** (*A*) Colonic weight-to-length ratio, (*B*) Hematoxylin and eosin stain of colon, (*C*) relative expression of Th1 cytokine IFNγ/TNFα and Th17 cytokine IL-17a/IL-17c in WT mice, *CD4-Cre*^+/Tg^*Mettl14*^FL/FL^ conditional knockout mice and *CD4-Cre*^+/Tg^*Mettl14*^FL/FL^ conditional knockout mice adoptively transferred with WT T_reg_ cells. Log_2_ scale was used on the y-axis. WT indicates wild-type littermate control. KO indicates *CD4-Cre*^+/Tg^*Mettl14*^FL/FL^ conditional knockout. Data are represented as mean ± SD; n = 4–5 mice per group. Data representative of 2 independent experiments with similar results.

To further test whether the colitis in *CD4-Cre*^+/Tg^ *Mettl14*^FL/FL^ conditional knockout mice was due to dysfunctional T_reg_ cells, we adoptively transferred WT T_reg_ cells into the *CD4-Cre*^+/Tg^ *Mettl14*^FL/FL^ conditional knockout mice to determine whether the colitis phenotype could be attenuated. We found that transferring WT T_reg_ cells into the *CD4-Cre*^+/Tg^ *Mettl14*^FL/FL^ conditional knockout mice attenuated the colitis phenotype as assessed by decreased colonic weight-to-length ratio (Figure 4*A*) and decreased inflammatory cell infiltration (Figure 4*B*) compared with untreated *CD4-Cre*^+/Tg^ *Mettl14*^FL/FL^ conditional knockout mice. Transferring WT T_reg_ cells into the *CD4-Cre*^+/Tg^ *Mettl14*^FL/FL^ conditional knockout mice also attenuated the levels of Th1 and Th17 cytokines compared with untreated *CD4-Cre*^+/Tg^ *Mettl14*^FL/FL^ conditional knockout mice (Figure 4*C*).

### *Mettl14*-Deficient T_reg_ Cells Have Decreased RORγt Expression

**Figure 5:**
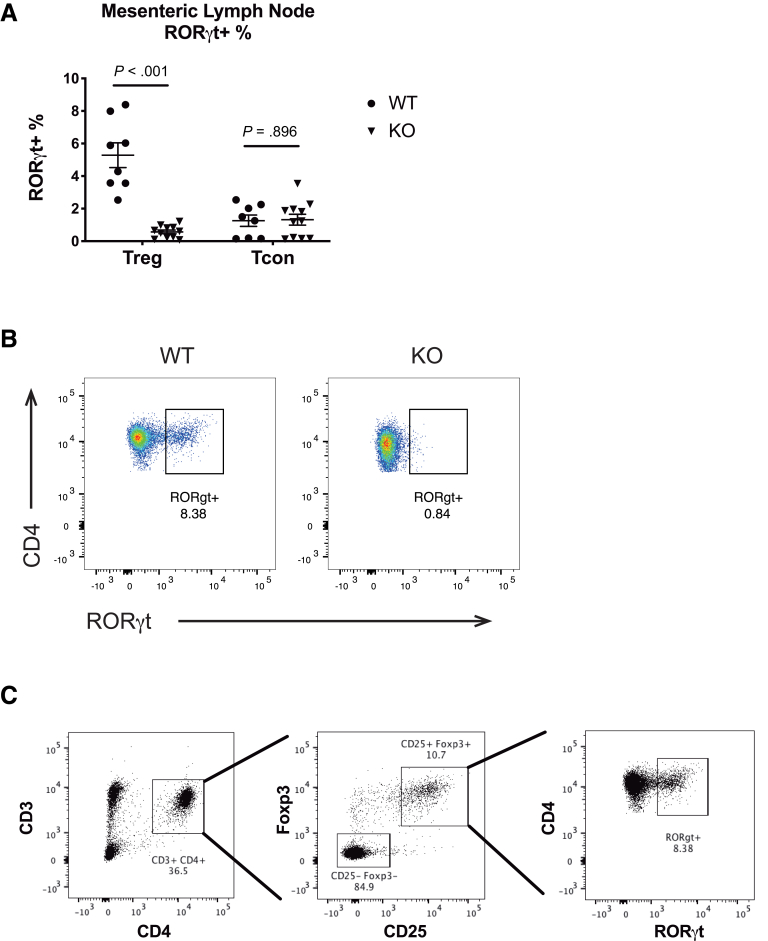
***Mettl14*-deficient T_reg_ cells have decreased RORγt expression.** (*A*) RORγt percentage in the mesenteric lymph nodes of T_reg_ and conventional T cells. (*B*) Representative flow cytometry image showing decreased RORγt expression in T_reg_ cells. The cells were gated from a parent population of CD25^+^Foxp3^+^ cells. (*C*) Gating strategy of flow cytometry analysis of RORγt^+^ cells. WT indicates wild-type littermate control. KO indicates *CD4-Cre*^+/Tg^*Mettl14*^FL/FL^ conditional knockout. Data are represented as mean ± SD; n = 8–11 mice per group. Combined data from 3 independent experiments shown.

The Foxp3^+^ RORγt^+^ T_reg_ cells have been shown to be a distinct stable subset of regulatory T cells that exhibit enhanced suppressive capacity in vivo.^19^ Because the *CD4-Cre*^+/Tg^ *Mettl14*^FL/FL^ mice have deficient T_reg_ cells, we asked whether the expression of RORγt was affected. Flow cytometry analysis of mesenteric lymph nodes showed that the *CD4-Cre*^+/Tg^ *Mettl14*^FL/FL^ conditional knockout mice had significantly reduced Foxp3^+^ RORγt^+^ T_reg_ cells compared with littermate control animals (Figure 5*A* and *B*). In contrast, the RORγt^+^ fraction is unchanged in conventional T cells (Figure 5*A*). The flow cytometry gating strategy is shown in Figure 5*C*.

### *Mettl14* Deficiency Causes Impaired Induction of Naïve T Cells Into Induced T_reg_ Cells

**Figure 6:**
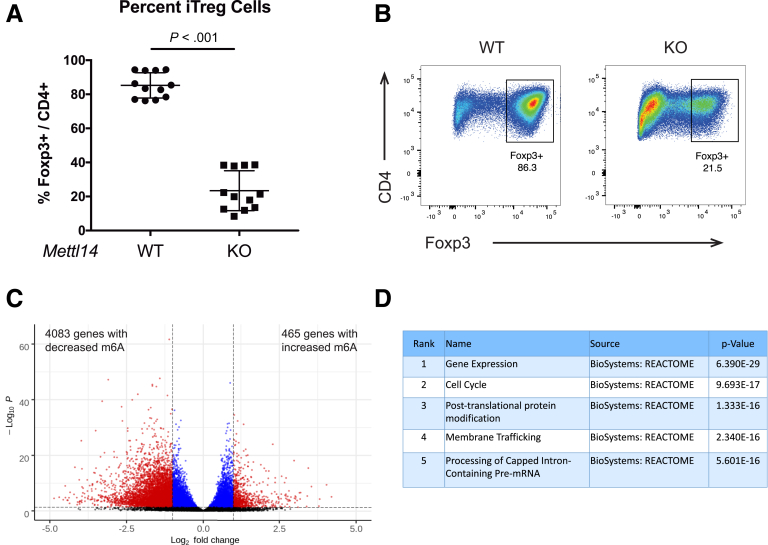
***Mettl14* deficiency causes impaired induction of naïve T cells into iT_reg_ cells.** (*A*) Induction efficiency of iT_reg_ cells from naïve T cells as evidenced by Foxp3 expression. (*B*) Representative flow cytometry image showing percent of Foxp3^+^ iT_reg_ cells after induction from naïve T cells. The cells were gated from a parent population of CD4^+^ cells. WT indicates wild-type littermate control. KO indicates *CD4-Cre*^+/Tg^*Mettl14*^FL/FL^ conditional knockout. Data are represented as mean ± SD; n = 12 per group. Combined data from 3 independent experiments shown. (*C*) m^6^A profiling of iT_reg_ cells compared with naïve T cells. Black color indicates nonsignificant; blue color indicates *P <* .05 and absolute Log_2_ fold change <1; red color indicates *P <* .05 and absolute Log_2_ fold change >1. (*D*) Significant pathways represented by genes with differential m^6^A levels. The top 5 most significant pathways were shown.

Induced T_reg_ (iT_reg_) cells has been reported to act synergistically with natural T_reg_ cells to control experimental colitis.^20^ We asked whether *Mettl14* deficiency affects induction of iT_reg_ from naïve T cells. While WT naïve T cells could be induced in vitro into iT_reg_ cells with 85% efficiency as evidenced by Foxp3 expression, *Mettl14*-deficient naïve T cells have a significantly reduced induction efficacy, with only 24% of cells expressing Foxp3 (Figure 6*A* and *B*). The m^6^A expression profile of WT iT_reg_ cells were compared with that of WT naïve T cells to determine genes with differential m^6^A levels. We found that there are 465 genes with increased m^6^A levels and 4083 genes with decreased m^6^A levels in iT_reg_ cells compared with naïve T cells (Figure 6*C*). Pathway analysis showed that the most significantly affected pathways include gene expression, cell cycle, and post-translational protein modification (Figure 6*D*).

### Antibiotics Treatment Attenuates the Colitis Phenotype in *CD4-Cre*^+/Tg^*Mettl14*^FL/FL^ Conditional Knockout Mice

**Figure 7:**
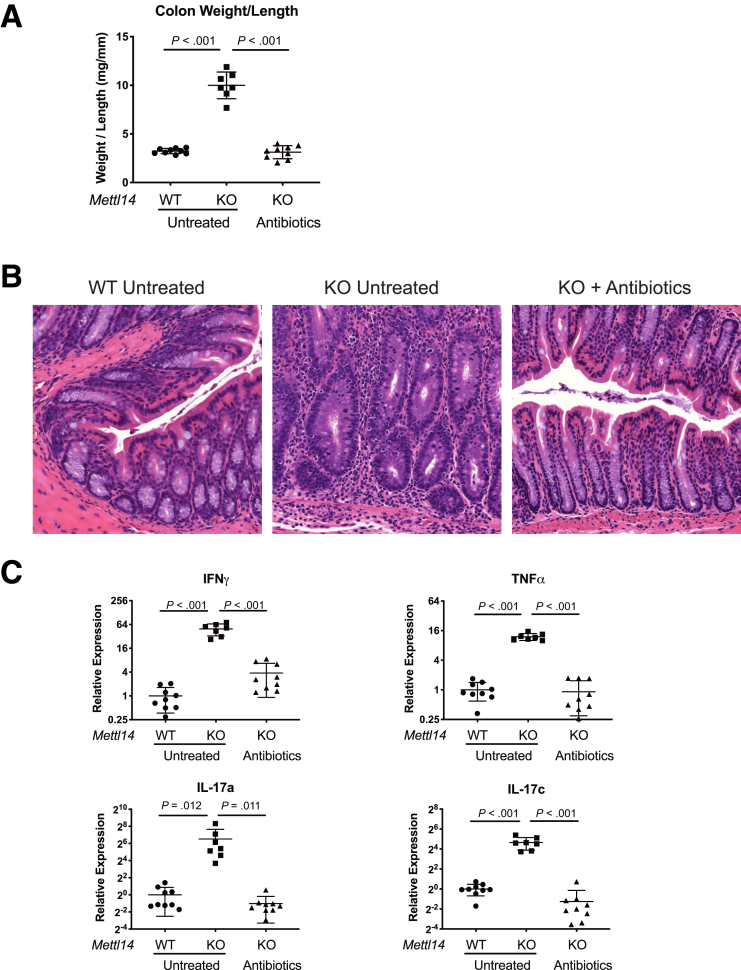

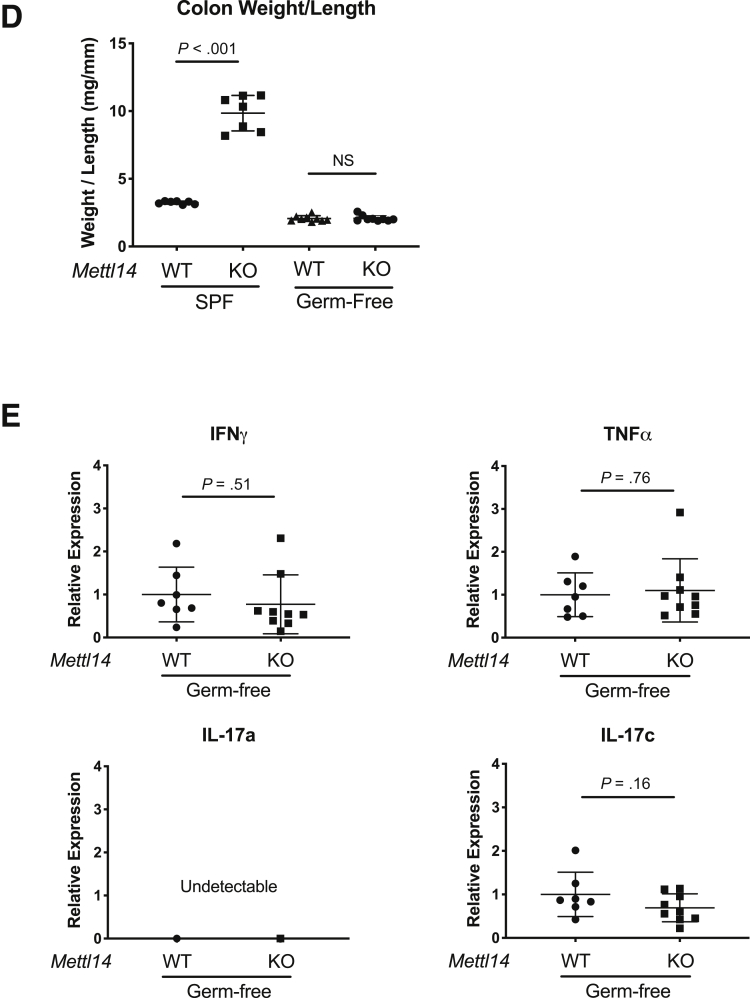
**Antibiotic treatment attenuates the colitis in *CD4-Cre*^+/Tg^*Mettl14*^FL/FL^ conditional knockout mice.** The *CD4-Cre*^+/Tg^*Mettl14*^FL/FL^ conditional knockout mice were treated with ciprofloxacin + metronidazole for 3 weeks and compared with untreated mice. (*A*) Colonic weight-to-length ratio, (*B*) hematoxylin and eosin stain of colon, and (*C*) relative expression of Th1 cytokine IFNγ/TNFα and Th17 cytokine IL-17a/IL-17c. Log_2_ scale was used on the y-axis. (*D*) Colonic weight-to-length ratio of mice in specific pathogen–free (SPF) facility compared with germ-free facility. (*E*) Relative expression of Th1 cytokine IFNγ/TNFα and Th17 cytokine IL-17a/IL-17c of mice in germ-free facility. WT indicates wild-type littermate control. KO indicates *CD4-Cre*^+/Tg^*Mettl14*^FL/FL^ conditional knockout. Data are represented as mean ± SD; n = 7–9 mice per group. Combined data from 2 independent experiments shown.

The colon is constantly exposed to microbial antigens. One of the functions of T_reg_ cells is to prevent inflammation induced by bacterial antigens and maintain homeostasis. Because we found that the colitis phenotype is likely due to dysfunctional T_reg_ cells in the *CD4-Cre*^+/Tg^ *Mettl14*^FL/FL^ conditional knockout mice, we determined whether the colitis phenotype could be attenuated by antibiotics treatment. We used a combination of ciprofloxacin and metronidazole which are the 2 antibiotics currently used for treatment of human IBD. We found that treating the *CD4-Cre*^+/Tg^ *Mettl14*^FL/FL^ conditional knockout mice with ciprofloxacin + metronidazole prevented the development of colitis phenotype as assessed by normal colonic weight-to-length ratio (Figure 7*A*) and eliminated inflammatory cell infiltration (Figure 7*B*). The antibiotic-treated *CD4-Cre*^+/Tg^ *Mettl14*^FL/FL^ conditional knockout mice also had normal levels of Th1 and Th17 cytokines (Figure 7*C*). In contrast, the untreated *CD4-Cre*^+/Tg^ *Mettl14* ^FL/FL^ conditional knockout mice developed colitis characterized by significantly increased colonic weight-to-length ratio (Figure 7*A*), inflammatory cell infiltration (Figure 7*B*), and elevated Th1 and Th17 cytokines (Figure 7*C*). We have further rederived the *CD4-Cre*^+/Tg^ *Mettl14*^FL/FL^ conditional knockout mice and WT littermate control mice in a germ-free environment. Under the germ-free environment, the *CD4-Cre*^+/Tg^ *Mettl14*^FL/FL^ conditional knockout mice did not develop colitis as evidenced by normal colonic weight-to-length ratio (Figure 7*D*) and normal cytokine expression profile (Figure 7*E*).

### Microbiome Analysis of the *CD4-Cre*^+/Tg^*Mettl14*^FL/FL^ Conditional Knockout Mice

**Figure 8:**
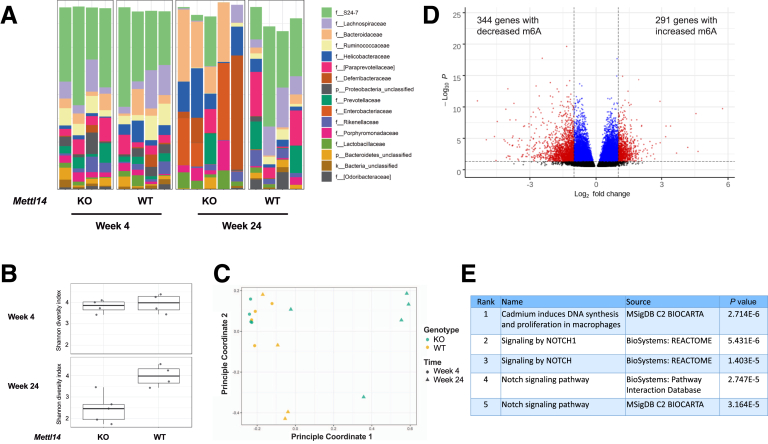
**Microbiome analysis of the *CD4-Cre*^+/Tg^*Mettl14*^FL/FL^ conditional knockout mice.** (*A*) Taxonomic composition, (*B*) alpha diversity, and (*C*) principal coordinate analysis plot of beta diversity of *CD4-Cre*^+/Tg^*Mettl14*^FL/FL^ conditional knockout mice compared with control animals at week 4 and week 24. WT indicates wild-type littermate control. KO indicates *CD4-Cre*^+/Tg^*Mettl14*^FL/FL^ conditional knockout. n = 4–5 mice per group. (*D*) m^6^A profiling of CD4^+^ T cells from germ-free mice compared with CD4^+^ T cells from specific-pathogen-free mice. Black color indicates nonsignificant; blue color indicates *P <* .05 and absolute Log_2_ fold change < 1; red color indicates *P <* .05 and absolute Log_2_ fold change > 1. (*E*) Significant pathways represented by genes with differential m^6^A levels. The top 5 most significant pathways were shown.

We analyzed the microbiome of the *CD4-Cre*^+/Tg^ *Mettl14*^FL/FL^ conditional knockout mice that had developed severe colitis and compared them to the microbiome of WT littermate control mice that do not have colitis. The *CD4-Cre*^+/Tg^ *Mettl14*^FL/FL^ conditional knockout mice and littermate control mice were housed together in the same cage to minimize cage differences. We found that at week 4, there was no difference in taxonomic composition between WT and *CD4-Cre*^+/Tg^ *Mettl14*^FL/FL^ conditional knockout mice (Figure 8*A*). At week 24, the taxonomic composition of the *CD4-Cre*^+/Tg^ *Mettl14*^FL/FL^ conditional knockout mice showed a reduction in the families S24-7 and *Lachnospiraceae*, and an increase in *Bacteroidaceae*, *Helicobacteraceae*, *Deferribacteraceae*, and *Enterobacteriaceae* compared with WT control animals (Figure 8*A*). The alpha diversity was substantially reduced at week 24 in the *CD4-Cre*^+/Tg^ *Mettl14*^FL/FL^ conditional knockout mice compared with WT control mice (Figure 8*B*). Principle coordinate analysis showed that there was a significant difference in beta diversity at week 24 in the *CD4-Cre*^+/Tg^ *Mettl14*^FL/FL^ conditional knockout mice compared with WT control mice (Figure 8*C*). Neither alpha diversity nor beta diversity was different at week 4 between the *CD4-Cre*^+/Tg^ *Mettl14*^FL/FL^ conditional knockout and WT control mice (Figure 8*B* and *C*). We additionally determined whether the microbiome affected m^6^A expression in CD4^+^ T cells. The m^6^A profile of CD4^+^ T cells isolated from germ-free mice were compared with that of CD4^+^ T cells isolated from mice from a specific pathogen–free facility. We found that there are 291 genes with increased m^6^A levels and 344 genes with decreased m^6^A levels in germ-free CD4^+^ T cells compared with T cells from specific pathogen free facility (Figure 8*D*). Pathway analysis showed NOTCH signaling as the most significantly affected pathway (Figure 8*E*).

## Discussion

Here, we show that deletion of RNA m^6^A methyltransferase component METTL14 in T cells leads to spontaneous colitis. The development of colitis is predominantly due to dysfunctional T_reg_ cells. The *Mettl14*-deficient T_reg_ cells were unable to suppress naïve T cell–induced inflammation in a T cell adoptive transfer model of colitis. By adoptively transferring WT T_reg_ cells into mice with *Mettl14*-deficient T cells, we were able to suppress the colitis development. We showed that *Mettl14* deficiency caused an impaired induction of naïve T cells into iT_reg_ cells. We also found that the colitis is dependent on the microbiome as we were able to suppress colitis development in the *CD4-Cre*^+/Tg^ *Mettl14*^FL/FL^ conditional knockout mice with antibiotic treatment. While there is a significant difference in microbiome between WT and *CD4-Cre*^+/Tg^ *Mettl14*^FL/FL^ conditional knockout mice at week 24, there is no difference in microbiome at week 4 before the development of colitis. The microbiome changes at week 24 may reflect alterations due to colonic inflammation.

One of the commonly used colitis models is the IL-10^–/–^ mice. The IL-10^–/–^ mice develop spontaneous colitis with a Th1 response.^21^ The development of colitis in the IL10^–/–^ mice is dependent on the genetic background and intestinal microbiota. It has been previously reported that in our facility the IL-10^–/–^ mice develops spontaneous colitis only 25%–30% of the time by week 24.^22^ Our colitis model with the *CD4-Cre*^+/Tg^ *Mettl14*^FL/FL^ conditional knockout mice has 100% penetrance with all mice developing early signs of colitis by week 6, and the colitis phenotype becomes progressively more severe over time. While the IL-10 model is ideal for testing pathobiont due to its incomplete penetrance, our colitis model is ideal for testing protective microbiota that could potentially attenuate colitis development. While we showed that antibiotics treatment induced remission in our colitis model, the role of antibiotics to induce or maintain remission in IBD is limited. This is a limitation of our knockout mouse model to reflect IBD pathogenesis.

Multiple other colitis models have been used to study IBD. Besides the IL-10^–/–^ mice, The most commonly used models include DSS (dextran sulfate sodium)-induced colitis, TNBS (2,4,6-trinitrobenzenesulfonic acid)-induced colitis, oxalazone-induced colitis, and T cell adoptive transfer colitis.^2^ Each of these models induce a different type of response and have specific advantages and disadvantages. DSS induces a colitis by disrupting the colonic epithelial barrier and is a useful model for studying epithelial injury, restitution, and innate immune mechanisms. TNBS is a hapten-carrier model that renders colonic proteins immunogenic with a Th1 predominant response, although the type of cytokine response is strongly strain-dependent. Oxalazone-induced colitis is also a hapten-carrier model, but it induces a Th2 predominant response.^2^ The T cell adoptive transfer colitis model is useful for studying T cell subpopulations transferred into immunodeficient mice.^18^ Our colitis model has several advantages including spontaneous rapid onset, 100% penetrance, progressively severe phenotype, and defined Th1/Th17-predominant cytokine profile. We have bred over 200 *CD4-Cre*^+/Tg^ *Mettl14*^FL/FL^ conditional knockout mice, and all of them showed weight loss and colitis by 4 months of age and scarified due to the development of colitis. We believe the our spontaneous colitis model will be a valuable tool to investigate the impact of impaired T cell function on gut homeostasis with specific focus on m^6^A methylation in regulation regulatory T cell function. In our model, the inflammation is in the colon and not the small bowel, and it may be a suitable model to study ulcerative colitis rather than Crohn’s disease, as ulcerative colitis has inflammation limited to the colon and Crohn’s disease frequently has both small and large bowel inflammation. While the *CD4-Cre*^+/Tg^ *Mettl14*^FL/FL^ conditional knockout mice do not seem to develop any other pathology, this does not rule out a possible immune deficiency–like phenotype. It would be of future interest to investigate whether this model would be suitable to study immune deficiency syndromes that primarily affect the gut.

The mRNA m^6^A methylation has been shown to be an important post-transcriptional gene regulation mechanism.^3^^,^^23^^,^^24^ This modification is added to the mRNAs by a methyltransferase complex with the catalytic core containing METTL3 and METTL14. It has previously been reported that lineage specific deletion of *Mettl3* in mouse T cells leads to chronic inflammation in the intestine after 3 months of age.^17^ This is similar to the phenotype we observed in our mice with lineage specific deletion of *Mettl14* in T cells, except that our mice developed colitis as early as week 6, and the inflammation became progressively more severe over time. Additionally, it was reported that mice with Foxp3-mediated deletion of *Mettl3* in regulatory T cells develop a severe systemic autoimmune response after weaning and start to die in 8–9 weeks.^17^ The regulatory T cells deficient in *Mettl3* lost their ability to suppress naïve T cell proliferation. In our colitis model, the deletion of *Mettl14* also leads to T_reg_ dysfunction with loss of suppressive capacity. While there also appears to be a dysfunction in the naïve T cells similar to that reported in the *Mettl3*-deficient naïve T cells,^14^ the T_reg_ dysfunction is predominant because the mice developed a proinflammatory colitis phenotype that could be reversed by adoptively transferring WT T_reg_ cells.

The causative role between intestinal microbiota and development of IBD is not yet established. However, multiple evidences support the gut microbiome’s involvement in IBD pathogenesis. Many studies have shown that patients with IBD have reduced bacterial diversity, but no causative microorganism has been consistently implicated to cause IBD. In patients with IBD that have their fecal stream diverted through a loop ileostomy, their colitis often improves.^25^ Patients with Crohn’s disease treated with antibiotics may experience a modest improvement in their disease activity.^26^ In patients with ulcerative colitis, an intensive multidonor fecal microbiota transplant over an 8-week period has been shown to improve their ulcerative colitis.^27^ The fecal microbiota transplantation was associated with a significantly increased fecal microbiota diversity.^27^ Prepared donor fecal microbiota transplantation has been shown to result in a higher likelihood of remission at 8 weeks compared with autologous fecal microbiota transplantation in patients with ulcerative colitis.^28^ In our colitis model, there is a decrease in both alpha and beta diversity associated with the development of colitis. The fecal microbiota are necessary for the development of colitis because the mice did not develop colitis when housed in a germ-free facility. We have additionally found that there are over 600 genes with differential m^6^A level in CD4^+^ T cells isolated from germ-free mice compared with CD4^+^ T cells isolated from specific pathogen–free mice. This shows that the gut microbiota can regulate the m^6^A epitranscriptome in CD4^+^ T cells. Similar results have been shown at the organ level in which the microbiome has been reported to affect m^6^A level in brain, intestine, and liver.^29^ The most significantly affected pathway is the NOTCH signaling pathway, which has been shown to regulate T cell homeostasis and differentiation.^30^ It is not clear whether the dysbiosis leads to the inflammation or is a secondary phenomenon. This would be an area of further investigation.

Thousands of mRNAs could be modified with m^6^A. It was previously reported that in the *Mettl3-*deficient regulatory T cells, the T_reg_ dysfunction is potentially mediated by increased SOCS level that suppresses the IL-2-Stat5 signaling pathway.^17^ There are likely additional pathways affected by the m^6^A modification that are important in maintaining T_reg_ function. One of the subsets of T_reg_ cells are the Foxp3^+^ RORγt^+^ T_reg_ cells that exhibit enhanced suppressive capacity in vivo.^19^ We have found that the *Mettl14*-deficient T_reg_ cells have decreased RORγt expression that likely contributes to their decreased suppressive capacity in vivo. The iT_reg_ cells have been shown to act synergistically with natural T_reg_ cells to control experimental colitis.^20^ We showed that Mettl14 deficiency causes impaired induction of iT_reg_ cells from naïve T cells in vitro. This could be a mechanism that contributes to the T_reg_ dysfunction seen in the *CD4-Cre*^+/Tg^ *Mettl14*^FL/FL^ conditional knockout mice. We found over 4000 genes with differential m^6^A expression in the iT_reg_ cells compared with naïve T cells. Significantly affected pathways include gene expression, cell cycle and post-translational protein modification. Other pathways affected by m^6^A modifications in T_reg_ cells would be interesting areas of future investigation.

In summary, we have identified a new spontaneous colitis model mediated by T cell deficiency of METTL14, a component of an m^6^A writer. The colitis development is dependent on dysfunctional T_reg_ cells and the intestinal microbiota. This model likely represents a new tool for elucidating pathogenic mechanisms, studying the contribution of intestinal microbiome and preclinical testing of therapeutic agents for IBD.

## Materials and Methods

### Mice

The generation of *Mettl14*^FL/FL^ mice have been described previously.^13^ The *Mettl14*^FL/FL^ mice were crossed with *CD4-Cre*^+/Tg^ mice (The Jackson Laboratory, Bar Harbor, ME) to generate *CD4-Cre*^+/Tg^ *Mettl14*^FL/FL^ conditional knockout mice and littermate control mice. The *Mettl14*^FL/FL^ mice were crossed with *Foxp3*^YFP–Cre^ mice (The Jackson Laboratory) to generate *Foxp3*^YFP–Cre^ *Mettl14*^FL/FL^ conditional knockout mice and littermate control mice. Littermate control animals were used for all experiments. Animals were housed under either specific pathogen–free conditions or in our gnotobiotic facility. The conditional knockout mice and littermate control mice were housed together to minimize cage differences in microbiome. The Institutional Animal Care and Use Committee of the University of Chicago approved the use of animals in these experiments.

### T Cell Transfer Colitis Model

The T cell transfer colitis model has been described previously.^18^ Briefly, for transfer of CD4^+^ naïve T cells, splenic CD4^+^ cells from *CD4-Cre*^+/Tg^ *Mettl14*^FL/FL^ conditional knockout mice and littermate control mice were isolated with CD4^+^ T cell isolation kit II (Miltenyi Biotec, Bergisch Gladbach, Germany) according to manufacturer’s protocol. The isolated CD4^+^ T cells were then stained with CD4-FITC, CD25-PE, CD62L-BV421 and CD44-APC (BioLegend, San Diego, CA). Naïve T cells were fluorescence-activated cell sorter (FACS) sorted as CD4^+^, CD25^–^, CD62L^+^, and CD44^–^ cells. Each recipient *Rag1*^–/–^ mice was injected with 5 × 10^5^ naïve T cells. For co-transfer of CD4^+^ naïve T cells and T_reg_ cells, naïve T cells were isolated as described previously from C57BL6-CD45.1 mice in which the CD45.1 marker can be used as a donor marker for naïve T cells. For isolation of T_reg_ cells, splenic CD4^+^ cells from *Foxp3*^YFP–Cre^ *Mettl14*^FL/FL^ conditional knockout mice and littermate control mice were isolated with CD4^+^ T cell isolation kit II (Miltenyi Biotec). The T cells were then stained with CD4-APC-Fire750 (BioLegend). T_reg_ cells were sorted as YFP^+^ CD4^+^ cells. Recipient *Rag1*^–/–^ mice were injected with naïve T cells and T_reg_ cells at a 2:1 ratio. The mice were then weighed every 3–4 days. Mice were scarified 7–8 weeks after T cell transfer.

### FACS Analysis

Mouse spleen or mesenteric lymph node was collected, and single-cell preparation was made by grinding the spleen or lymph node between 2 glass slides. Red blood cell lysis was performed with ACK (ammonium-chloride-potassium) lysing buffer according to manufacturer’s protocol (Thermo Fisher Scientific, Waltham, MA). Cells are filtered with a 70-μm filter and stained for FACS analysis according to previously published protocols.^31^ The CD3-BUV395, CD45.2-PE-CF594, and RORγt-BV421 antibodies are from BD Biosciences (Franklin Lakes, NJ). The CD4-FITC, CD25-PE, CD62L-BV421, CD44-APC, CD45.1-BUV785 antibodies are from BioLegend.

### Histology

Mouse tissues were collected and fixed in 10% neutral buffered formalin (Thermo Fisher Scientific). Tissues were embedded in paraffin and stained with hematoxylin and eosin.

### Quantitative Reverse-Transcription Polymerase Chain Reaction

Total RNA was reverse transcribed using the High Capacity cDNA Reverse Transcription kit (Thermo Fisher Scientific). Samples were analyzed by TaqMan quantitative reverse-transcription polymerase chain reaction. TaqMan gene expression master mix and all primer/probe sets were obtained from Thermo Fisher Scientific. Relative expression was calculated using the comparative C_T_ method.^32^

### Antibiotics Treatment

A combination of ciprofloxacin (200 mg/L) and metronidazole (600 mg/L) was added to drinking water of *CD4-Cre*^+/Tg^ *Mettl14*^FL/FL^ conditional knockout mice. The antibiotics doses have previously been shown to attenuate experimental colitis in mice.^33^

### Adoptive Transfer of T_reg_ Cells

T_reg_ cells were isolated by FACS sorting from Foxp3^YFP^ mice based on YFP expression. The *CD4-Cre*^+/Tg^ *Mettl14*^FL/FL^ conditional knockout mice were adoptively transferred 1 × 10^6^ T_reg_ cells on day 0, day 7, and day 14. Control mice were injected with phosphate-buffered saline on these days. Mice were sacrificed at day 25.

### Culture and FACS Analysis of iT_reg_ Cells

Culture of iT_reg_ cells were performed according to previously described protocol.^34^ Briefly, CD4^+^ naïve T cells were cultured in Advanced RPMI 1640 + 10% fetal bovine serum + 1% penicillin/streptomycin + 2-mM L-glutamine + 20-ng/mL transforming growth factor beta + 40-ng/mL IL-2 + 1-nM all-trans retinoic acid. Cells were collected after 96 hours of culture. Foxp3 expression was analyzed by intracellular FACS staining with eBioscience mouse regulatory T cell staining kit 1 (Thermo Fisher Scientific) according to manufacturer’s protocol.

### m^6^A Sequencing and Analysis

m^6^A sequencing was performed according to previously published protocol.^35^ RNA was extracted by TRIZOL reagent and Direct-zol RNA miniprep (Zymo Research, Irvine, CA). PolyA mRNA was enriched with a Dynabeads mRNA DIRECT Purification Kit (Thermo Fisher Scientific). RNA fragmentation was performed with Bioruptor Pico sonicator (Diagenode, Denville, NJ). m6A-IP was performed using EpiMark *N*^6^-Methyladenosine Enrichment Kit (New England Biolabs, Ipswich, MA). Library preparation was done with SMARTer Stranded Total RNA-Seq Kit v2 (Takara, Kyoto, Japan). Sequencing was performed by the University of Chicago Genomics Facility on an Illumina HiSeq4000 (Illumina, San Diego, CA). For data analysis, reads were mapped to the mouse genome and transcriptome mm10 using HISAT (version 2.1.0).^36^ m^6^A peaks in the mapped reads were called by RADAR.^37^ Parameters of *P* value <.05 and absolute (log_2_ fold change) >1 was applied to call significant peaks. Pathway analysis was performed using the ToppGene suite (toppgene.cchmc.org).^38^ The sequencing data was deposited into the Gene Expression Omnibus database (Accession number: GSE152234).

### Microbiome Analysis

Fecal pellets were placed into a MP Bio PowerMag Soil DNA Isolation Bead Plate (MP Bio, Santa Ana, CA). DNA was extracted following MP Bio’s instructions on a KingFisher robot. Bacterial 16S rRNA genes were polymerase chain reaction–amplified with dual-barcoded primers targeting the V4 region.^39^ Amplicons were sequenced with an Illumina MiSeq using the 300-bp paired-end kit. Sequences were denoised, taxonomically classified using Greengenes (v. 13.8) as the reference database, and clustered into operational taxonomic units (OTUs) with 97% similarity using the Mothur software package (v. 1.39.5).^40^ Alpha diversity was estimated with the Shannon index on raw OTU abundance tables. Differences in the significance of diversity were tested with an analysis of variance. To estimate beta diversity across samples, we excluded OTUs occurring in fewer than 10% of the samples with a count of <3 and computed Bray-Curtis indices. We visualized beta diversity, emphasizing differences across samples, using principal coordinate analysis ordination. All analyses were conducted in the R environment (v. 3.5.1) (R Foundation for Statistical Computing, Vienna, Austria). The DNA extraction and microbiome analysis were performed by Microbiome Insights, Inc (Vancouver, Canada).

### Statistical Analysis

Statistical analyses were performed using GraphPad Prism 7.0 (GraphPad Software, San Diego, CA). Student’s *t* test and 1-way analysis of variance were used where appropriate. *P* values <.05 were considered statistically significant. All authors had access to the study data and had reviewed and approved the final manuscript.
